# A Genome-Wide Association Analysis Identified a Novel Susceptible Locus for Pathological Myopia at 11q24.1

**DOI:** 10.1371/journal.pgen.1000660

**Published:** 2009-09-25

**Authors:** Hideo Nakanishi, Ryo Yamada, Norimoto Gotoh, Hisako Hayashi, Kenji Yamashiro, Noriaki Shimada, Kyoko Ohno-Matsui, Manabu Mochizuki, Masaaki Saito, Tomohiro Iida, Keitaro Matsuo, Kazuo Tajima, Nagahisa Yoshimura, Fumihiko Matsuda

**Affiliations:** 1 Department of Ophthalmology and Visual Sciences, Kyoto University Graduate School of Medicine, Kyoto, Japan; 2 Center for Genomic Medicine, Kyoto University Graduate School of Medicine, Kyoto, Japan; 3 Human Genome Center, Institute of Medical Science, University of Tokyo, Tokyo, Japan; 4 Department of Ophthalmology and Visual Science, Tokyo Medical and Dental University Graduate School of Medicine, Tokyo, Japan; 5 Department of Ophthalmology, Fukushima Medical University, Fukushima, Japan; 6 Division of Epidemiology and Prevention, Aichi Cancer Center Research Institute, Nagoya, Japan; 7 Aichi Cancer Center Research Institute, Nagoya, Japan; 8 CEA/Institute de Genomique, Centre National de Génotypage, Evry, France; National Institute of Genetics, Japan

## Abstract

Myopia is one of the most common ocular disorders worldwide. Pathological myopia, also called high myopia, comprises 1% to 5% of the general population and is one of the leading causes of legal blindness in developed countries. To identify genetic determinants associated with pathological myopia in Japanese, we conducted a genome-wide association study, analyzing 411,777 SNPs with 830 cases and 1,911 general population controls in a two-stage design (297 cases and 934 controls in the first stage and 533 cases and 977 controls in the second stage). We selected 22 SNPs that showed *P*-values smaller than 10^−4^ in the first stage and tested them for association in the second stage. The meta-analysis combining the first and second stages identified an SNP, rs577948, at chromosome 11q24.1, which was associated with the disease (*P* = 2.22×10^−7^ and OR of 1.37 with 95% confidence interval: 1.21–1.54). Two genes, *BLID* and *LOC399959*, were identified within a 200-kb DNA encompassing rs577948. RT–PCR analysis demonstrated that both genes were expressed in human retinal tissue. Our results strongly suggest that the region at 11q24.1 is a novel susceptibility locus for pathological myopia in Japanese.

## Author Summary

Myopia is one of the most common ocular disorders with elongation of axis of the eyeball. Pathological myopia or high myopia, a subset of myopia which is characterized with excessive axial elongation and degenerative changes of the eye, is a leading cause of visual impairment. Since genetic factors play significant roles in its development, identification of genetic determinants is an urgent and important issue. Although family-based linkage analyses have isolated at least 16 susceptible chromosomal loci for pathological or common myopia, no gene responsible for the disease has been identified. We conducted the first genome-wide case/control association study of pathological myopia in a two-stage design using 411,777 markers with 830 Japanese patients and 1,911 Japanese controls. We identified a region strongly suggestive for the disease susceptibility at chromosome 11q24.1 containing *BLID* and *LOC399959*. Their expression was confirmed in human retina with RT–PCR. *BLID* encodes an inducer of apoptotic cell death, and apoptosis is known to play an important functional role in pathological myopia. We believe that our study contributes to further dissect the molecular events underlying the development and progression of pathological myopia.

## Introduction

Myopia is a refractive error (http://en.wikipedia.org/wiki/Refractive_error) of the eye in which parallel rays of light focus in a plane anterior to the retina resulting in blurred vision. Myopia is one of the most common ocular disorders worldwide, and is in much higher prevalence in Asians than in Caucasians. Recent population-based surveys in the elderly reported that the prevalence of myopia was approximately 25% in the Caucasian populations [1] and 40% in the East Asian (Chinese and Japanese) populations [2],[3].

Myopia is divided into two distinct subsets, namely, common and pathological myopia. Pathological myopia, also called high myopia, is distinguished from common myopia, also called low/moderate myopia, by excessive increase in axial length of the eyeball, which is the most important contributor to the myopic refraction [4],[5]. The axial length of the eyeball in adults is approximately 24 mm, and its elongation by 1 mm without other compensatory changes results in a myopic shift of −2.5 to −3.0 diopters (D). It has been shown that distribution of the axial lengths of the adult myopic population is bimodal [6], and the subgroup with elongated axial length in the bimodal distribution corresponds to pathological myopia. This group comprises 1% to 5% of the population [3],[7], and is commonly defined by axial length greater than 26.0 mm which is equivalent to refractive errors greater than −6 D [8].

The excessive elongation of the eyeball causes mechanical strain with subsequent degenerative changes of the retina, choroid, and sclera. The degenerative changes at the posterior pole of the eye such as chorioretinal atrophy or posterior staphyloma are clinically important and unique to pathological myopia [9]. These unique degenerative changes at the posterior pole result in uncorrectable visual impairment due to decreased central vision and make pathological myopia one of the leading causes of legal blindness in developed countries [10]–[13].

It has been reported that not only environmental factors, such as near work and higher education, but also genetic factors contribute to the development of myopia, in particular, of pathological myopia [14]. Previous twin studies reported that the estimated heritability of refractive error and axial length is up to 0.90 [15],[16], although that might be overestimated due to common environmental effects [17]. Multiple family-based whole genome linkage analyses of myopia reported at least 16 susceptible chromosomal loci (MYP1–16 in OMIM database; 10 loci for pathological myopia [18]–[27] and 6 for common myopia [28]–[30]). Among them, at least 8 chromosomal loci, such as 12q21–23 (MYP3), 22q12 (MYP6) and 2q37.1 (MYP12) were successfully validated by at least two independent studies [31],[32]. However, no genes responsible for the disease have been identified.

The genome-wide association (GWA) study using single nucleotide polymorphisms (SNPs) as markers is an alternative approach to identify genetic risk factors of common diseases. This approach has been successfully applied to identify genetic risk factors for multigenetic diseases including ophthalmic diseases such as age-related macular degeneration [33],[34] and exfoliation syndrome [35]. To identify the genetic risk factors of pathological myopia, we conducted a two-stage GWA-based case/control association analysis using 411,777 markers with 830 Japanese patients and 1,911 Japanese controls (297 cases and 934 controls in the first stage, and 533 cases and 977 controls in the second stage).

## Results

### Characterization of the patients with pathological myopia

A total of 839 pathological myopic patients with axial length greater than 26.0 mm in both eyes were enrolled in the current study. In order to maximize the detection power, patients with axial length greater than 28.0 mm in both eyes were enrolled in the first stage of genome scan. No other clinical features were accounted for the assignment of patients to either stage. 824 out of 839 patients (98.2%) had degenerative changes specific to pathological myopia. Other features of cases and controls who passed quality control procedures of genotyping results (see Materials and Methods) were summarized in Table 1.

**Table 1:** Characteristics of the study population used in the study^a^.

Cases/Controls	Category	Subcategory	First stage	Second stage
**Cases: Patients with pathological myopia**	Number		297	533
	Age (years)		58.8±13.2	59.0±14.3
	Gender	Male	93	171
		Female	204	362
	Axial length (mm)	Right eyes	29.97±1.36	29.04±1.97
		Left eyes	29.84±1.37	28.91±1.89
	Refraction of the phakic eyes (Diopter)^b^	Right eyes	−14.94±4.04	−12.40±4.48
		Left eyes	−14.64±3.98	−12.07±4.72
**Controls: general Japanese population**	Number		934	977
	Age (years)		NA	48.3±16.3
	Gender	Male	NA	497
		Female	NA	480

The ± sign is a standard deviation.

a The study population after quality control procedures.

b For the calculations of refraction, 177 eyes (29.8%) in the first stage and 303 eyes (28.4%) in the second stage that had undergone cataract surgery or corneal refractive surgery were excluded.

### Genome-wide association analysis

**Figure 1:**
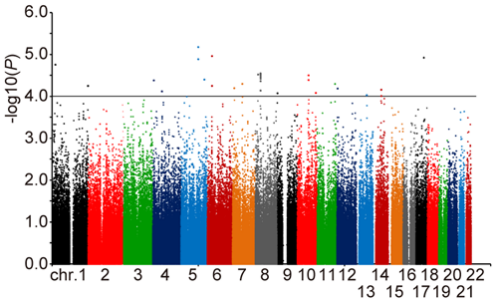
Manhattan plot of the first stage results for pathological myopia. Adjusted *P*-values obtained by the trend χ^2^ test for 411,777 SNPs on autosomes in 297 pathological myopic cases and 934 general population controls are plotted in −log_10_ scale according to their chromosome location.

For the first stage, we scanned the genome of 302 cases using the Illumina HumanHap550 BeadChip, which launches 561,466 relatively frequent SNPs (minor allele frequency>0.05) distributed across the human genome at an average interval of 6.5 kilobases (kb). Five cases and 149,689 SNPs were excluded due to quality control criteria (see details in Materials and Methods) and genotyping results of 411,777 SNPs in autosomes for 297 cases were used for the statistical analysis. They were compared with 934 controls from the JSNP database [36] for association with phenotype using χ^2^ test for trend. Genomic Control (GC) method [37] revealed only a slight inflation of the test statistics (GC parameter λ = 1.068). We identified 29 SNPs in 22 chromosomal regions with *P*-value adjusted by GC being smaller than 10^−4^ (Figure 1 and Table S1). Among them, seven SNPs at chromosome 8p12 were in strong linkage disequilibrium (LD) and likewise two SNPs at chromosome 10q22.2 (pair-wise D′>0.95 and r^2^>0.9). Thus, we selected one representing SNP from each region and tested 22 SNPs in the second stage.

For the second stage analysis, 537 cases and 980 population controls were genotyped by Taqman method. Among them, four cases and three controls were excluded due to low call rates (<90%). Genotyping success rates of the 22 SNP markers in the remaining 1,510 samples were greater than 96.8%. The genotype counts of the first and second stages were combined for meta-analysis. One SNP, rs577948, showed a strongly suggestive association (*P* = 2.22×10^−7^) (Table 2) in the meta-analysis whereas the remaining 21 SNPs were not significant (*P*>10^−5^) (Table S1).

**Table 2:** Association of SNP markers within the linkage disequilibrium block on chromosome 11q24.1 with pathological myopia in Japanese population.

				Meta-analysis^c^		First stage (N = 1,231)				Second stage (N = 1,510)			
SNP ID	Position^a^	Ref.^b^	Var.^b^	*P*-value	OR (95%CI)^d^	Ref. allele freq.		Nominal *P*	OR (95%CI)^d^	Ref. allele freq.		Nominal *P*	OR (95%CI)^d^
						Case (N = 297)	Control (N = 934)			Case (N = 533)	Control (N = 977)		
rs577948	121535400	A	G*	2.22×10^−7^	1.37 (1.21–1.54)	0.40	0.50	2.80×10^−5^	1.50 (1.24–1.81)	0.42	0.48	1.42×10^−3^	1.29 (1.11–1.50)
rs11218544	121544262	T*	G	5.48×10^−6^	1.33 (1.18–1.51)	0.70	0.61	7.90×10^−5^	1.50 (1.23–1.83)	0.66	0.61	8.94×10^−3^	1.24 (1.06–1.44)
rs10892819	121579254	T	G*	0.04	1.15 (1.01–1.31)	0.69	0.75	2.98×10^−3^	1.36 (1.11–1.67)	0.72	0.73	0.74	1.03 (0.87–1.22)
rs11218553	121590345	A	G*	8.28×10^−3^	1.18 (1.04–1.34)	0.60	0.67	1.77×10^−3^	1.36 (1.12–1.65)	0.66	0.68	0.39	1.07 (0.91–1.26)

a The position of markers on chromosome 11 refers to NCBI Build 36.1.

b Ref. and Var. are the reference and variant nucleotides, respectively, that are defined on the reference sequence of NCBI Build 36.1.

c Statistical results using the Mantel-Haenzel method as a fixed-effect model were shown.

d Odds ratios (ORs) were calculated for the causative allele (indicated with an asterisk).

### Evaluation of the region with rs577948

**Figure 2:**
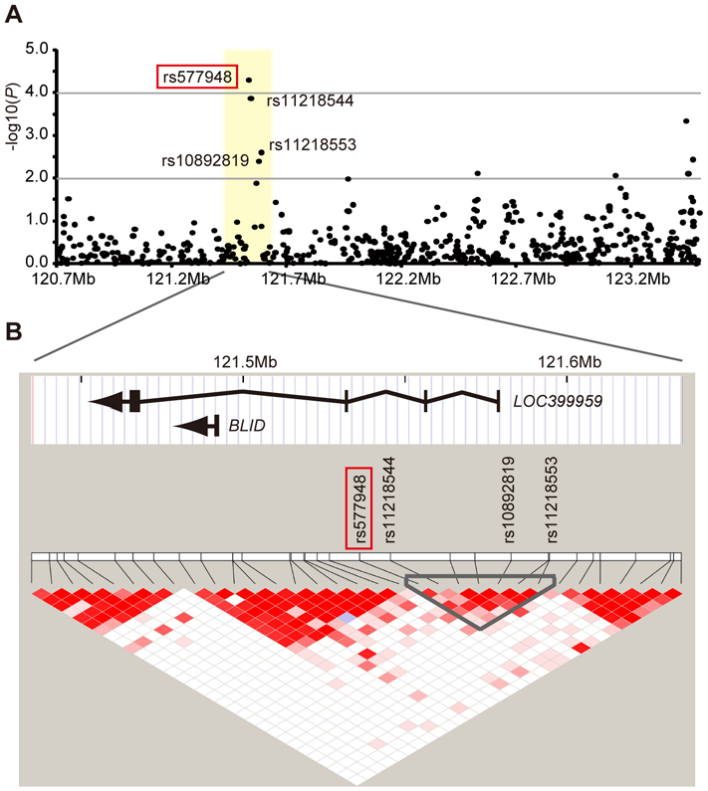
Results of genome scan at 11q24.1 locus containing the *BLID* and *LOC399959* genes. (A) Adjusted *P*-values on −log_10_ scale for SNPs examined for their association by the trend χ^2^ test. (B) Structures, orientations and locations of the *BLID* and *LOC399959* genes on NCBI Reference Sequence Build 36.1, together with pair-wise LD estimates of the SNP markers located within a 200-kb region encompassing the rs577948 marker (red box). Three additional SNP markers (rs11218544, rs11218553, and rs10892819), that showed adjusted *P*-value<10^−2^ in the first stage, are also indicated.

The SNP rs577948 which showed *P* = 2.22×10^−7^ by meta-analysis with OR of 1.37 (95% confidence interval (CI): 1.21–1.54) for the risk allele (nominal *P* = 2.80×10^−5^ and *P* = 1.42×10^−3^ in the first and second stages, respectively) (Table 2) was located at chromosome 11q24.1 (Figure 2A). Using the results of the first stage, an LD block which extended a 55-kb region containing rs577948 was generated. Six additional SNP markers within the block were included in the genome scan chip (Figure 2B). Among them, we selected three markers with adjusted *P*-value smaller than 0.01 in the first stage for further genotyping by Taqman method with DNAs used for the second stage. Weaker associations than that of rs577948 were obtained for these three markers by meta-analysis (Table 2). As shown in Figure 2B, two genes were located in a 200-kb region containing rs577948. *BLID* is a cell death inducer containing BH3-like motif [38], which is located approximately 44-kb upstream of rs577948. The other gene, *LOC399959*, is a hypothetical non-coding RNA [39] which encompassed 114-kb DNA in the region, and rs577948 is located in its second intron.

### Expression of the *BLID* and *LOC399959*

**Figure 3:**
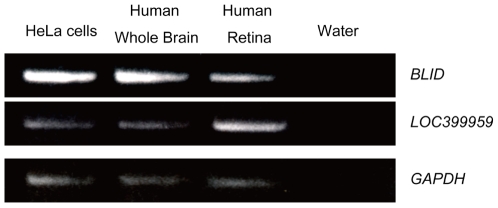
Expression of the *BLID* and *LOC399959* genes in the human retina. RT–PCR analyses of *BLID* and *LOC399959* expression in HeLa cells, the human Brain and the human retina. *GAPDH* was used as an internal control for cDNA quantification.

*BLID* is known as a cell-death inducer expressed in cytoplasm, in mitochondria at lower abundance, and in various human cancer cells from different tissues [38]. *LOC399959* was reported as a hypothetical non-coding RNA with a relatively ubiquitous expression pattern. We assessed the expression of the genes by RT-PCR using cDNAs of human retina and brain and those of HeLa cells as positive control. Expressions of both genes were detected in human retinal tissue as well as in human brain and HeLa cells (Figure 3).

## Discussion

Myopic refraction and axial length are reported to be a complex trait under polygenic control in which contribution of each gene is relatively small [40]. In the current study, two-stage GWA analysis identified a region at chromosome 11q24.1, in which rs577948 showed strongly suggestive *P* = 2.22×10^−7^ with OR of 1.37 (95% CI: 1.21–1.54) for the allele G.

Our GWA study identified only one strongly-suggestive locus. This may principally be due to the sample size of our study not being adequate. Recent genetic studies of complex traits with higher prevalence enroll much larger number of samples. In contrast, recruitment of patients with pathological myopia is difficult due to its lower prevalence, particularly those with degenerative changes (namely degenerative myopia). In order to improve insufficient detection power, we assigned pathological myopia patients with longer axis (greater than 28.0 mm) to the first stage. This strategy might be the reason we were successful in identifying the candidate region with relatively small number of cases.

Insufficiency of detection power due to a limitation in sample number may be a reason for difference between the findings of preceding linkage studies and ours. OMIM database lists 10 MYP regions (MYP1–5, 11–13, 15 and 16) for pathological myopia [18]–[27] and 6 MYP regions (MYP6–10 and 14) for common myopia [28]–[30]. None of these 16 MYPs are on chromosome 11q. Stambolian and colleagues reported heterogeneity LOD score of 1.24 at 11q23 in their linkage study for common myopia in Ashkenazi Jewish descent, which is the closest locus to our region reported to date [29]. Because the linkage signal was not strong and the band 11q23 (chr11, position 110,000 kb to 120,700 kb in the NCBI database) is more than 800 kilobases apart from our LD block in 11q24.1 (chr11, position 121,535 kb to 121,590 kb), whether or not they overlap each other is inconclusive. On the other hand, our study did not identify the associated SNPs in any of MYPs.

Although the insufficiency of detection power may be a reason for difference between our study and the linkage studies, there are other possible reasons. In general, any difference in the study designs could cause heterogeneous results. Firstly, there are two definitions of pathological myopia based on two distinct criteria, namely, the axial length and refractive error. In the current study, we enrolled pathological myopic patients based on the axial length (greater than 26.0 mm in both eyes), and not on the refractive error commonly used in the previous studies (refractive errors greater than −6 D). We focused on patients with vision-threatening degenerative changes [9] and the axial length fits better than refractive error for our purpose. The mean refraction in our myopic patients was −13.14±4.57 D (eyes that had undergone cataract surgery or corneal refractive surgery were excluded from this calculation) which indeed correspond to pathological myopic group in the previous linkage studies. On the other hand, it is not clear whether the patients enrolled in the linkage studies fulfill our criteria because the distribution of axial length and degenerative phenotypes in the cases are unknown. The difference in definition of pathological myopia may result in different susceptibility loci between studies.

Secondly, the methodology used is different between studies, namely, linkage analysis and association analysis using linkage disequilibrium mapping. The results of linkage and association studies of complex genetic traits are often different. Family-based linkage analysis is much more suitable for identifying rare genetic variants with large effects whereas SNP-based GWA analysis is more powerful in detection of relatively common variants with smaller effects in complex diseases [41].

Finally, the difference can also be due to the ethnicities of the samples enrolled. In the current study, all cases and controls were Japanese. Only one genome-wide linkage study has previously been published for pathological myopia in Japanese [42] and the others were for non-Japanese populations.

It would be interesting and important to examine the association of our locus in other ethnicities. Ethnic variations in disease susceptibility genes have been reported in various genetic traits including ophthalmological disorders. One such example is an SNP in the complement factor H gene (rs1061170) which has a large effect size with age-related macular degeneration in Caucasians [33],[43],[44] but much smaller in East Asian populations due to a remarkably lower risk allele frequency (∼35% in Caucasians and ∼5% in East Asians) [45]. Another example is exfoliation syndrome and *LOXL1* where the risk allele of rs1048661 is inverted between Icelandic (allele G) and Japanese (allele T) populations [35],[46]. Because of a large variation in prevalence of myopia among ethnic groups, a future trans-ethnic investigation of myopia risk genes will be important to dissect genetic backgrounds underlying the etiology of myopia.

Although the susceptibility locus contains *BLID* and *LOC399959*, it seems premature to discuss the involvement of *LOC399959* in myopia since it is a hypothetical non-coding gene. *BLID* plays a proapoptotic role involving the BH3-like domain by inducing a caspase-dependent mitochondrial cell death pathway [38]. Indeed, several animal and pathological studies suggested the functional role of apoptosis in pathological myopia [47],[48]. Moreover, a recent genome-wide linkage study followed by a fine-scale association mapping identified a myopia susceptibility gene locus containing the *PARL* gene which inhibits the mitochondrial pathway of apoptosis by interaction with *OPA1* [49]. In this context, *BLID* seems functionally relevant with the pathogenesis of pathological myopia. However, the true functional origin of association in this region has yet to be determined by further detailed investigation along with replication studies to validate our findings.

## Materials and Methods

### Study subjects

All procedures used in this study conformed to the tenets of the Declaration of Helsinki. The Institutional Review Board and the Ethics Committee of each institution approved the protocols used. All the participants were fully informed of the purpose and procedures, and a written consent was obtained from each.

Japanese pathological myopic cases were recruited at the Center for Macular Diseases of Kyoto University Hospital, the High Myopia Clinic of Tokyo Medical and Dental University, and Fukushima Medical University Hospital. All subjects underwent comprehensive ophthalmologic examinations, including dilated indirect and contact lens slit-lamp biomicroscopy, automatic objective refraction evaluation, and measurement of the axial length by applanation A-scan ultrasonography (UD-6000, Tomey, Nagoya, Japan) or partial coherence interferometry (IOLMaster, Carl Zeiss Meditec, Dublin, CA).

As a general population control of the first stage, genotype count data of 934 healthy Japanese subjects were obtained from the JSNP database [36]. For the second stage, 980 healthy Japanese individuals were recruited at Aichi Cancer Center Research Institute. Genomic DNAs were extracted from peripheral blood leukocytes with QuickGene-610L DNA extraction kit (FUJIFILM Co., Tokyo, Japan).

### Genome-wide association analysis

We designed to scan the genome in two stages. A total of 839 patients and 1,914 controls were separated into two groups; 302 cases and 934 controls for the first stage, and 537 cases and 980 controls for the second stage. In order to increase the detection power, patients with longer axis of the eyeball (greater than 28.0 mm) were principally assigned to the first stage.

For the first stage analysis, 561,466 SNPs were genotyped in 302 patients of pathological myopia using Illumina HumanHap550 chips (Illumina Inc., San Diego, CA). This chip covers approximately 87% of the common genetic variations in the Asian population [50]. Cluster definition for each SNP was performed using Illumina BeadStudio Genotyping Module. A systematic quality control procedure of the genome scan results was applied as follows. Samples were evaluated for data quality first and markers were subsequently excluded. Genetic proximity of sample pairs was evaluated with pi-hat in PLINK [51] and four samples with indication of kinship or sample duplication were excluded. Genotypes in X chromosome were used for checking the precision of the phenotype record, and only one sample was removed due to mismatch in gender. The final sample size of pathological myopia was 297. As a population-based control, genotype count data by the genome scanning of 934 healthy Japanese subjects using the same chip were obtained from the JSNP database [36]. The chip contained 515,154 markers in autosomes that are common in the cases and controls. We excluded 78 SNPs due to low successful call rate (<95%) in the cases, 1,760 SNPs due to the distortion of Hardy-Weinberg Equilibrium (HWE) in the controls (*P*<10^−3^ by HWE exact test) and 46,722 monomorphic SNPs. 54,817 SNPs with minor allele frequency less than 0.05 in both cases and controls were also excluded. After these quality control procedures, a total of 411,777 SNPs were used for the statistical analysis. The genotyping call rate was greater than 97.43% (median call rate 99.99%) for DNA sample and 98.21% (median call rate 100%) for SNP marker.

Association between genotypic distribution of each SNP and the disease was examined using a χ^2^ test for trend. The OR and the 95% CI were estimated using Woolf's method [52]. Inflation in the test statistics was assessed using the genomic-control method [37]. Haploview [53] software was used to infer the LD in the targeted regions. SNPs with *P*-value adjusted by genomic control being smaller than 10^−4^ were selected as candidates for second stage. Among the candidate SNPs, LD indices (D′ and r^2^) were calculated with Haploview and when multiple SNPs were in strong LD (D′>0.95 and r^2^>0.9), one representative SNP was chosen to be genotyped in the second stage.

In the second stage, 537 cases and 980 controls were genotyped with the Taqman SNP assay using the ABI PRISM 7700 system (Applied Biosystems, Foster City, CA). The 302 pathological myopic cases in the first stage were also genotyped to validate the concordance between Illumina Infinium assay and Taqman assay. Samples with low successful call rate (<90%) were excluded from the study. Subsequently four cases and three controls were excluded and data of 533 cases and 977 controls were used for the analysis. The concordance rate ranged between 98.68% and 100% for the 22 SNPs. The genotype counts of the first and second stages were combined for meta-analysis using the Mantel-Haenzel method [54] as a fixed-effect model. The OR heterogeneity between the first stage and the second stage was evaluated using Cochran's Q-statistic *P*-value. The data from the second stage were also evaluated for association independently from the first stage.

### Screening for *BLID* and *LOC399959* expression

Human retina cDNAs were obtained from Takara Bio Inc. (Kyoto, Japan). Total RNA of HeLa cells and human whole brain were also obtained from the same manufacturer and cDNAs were synthesized using the First-Strand cDNA Synthesis Kit (GE Healthcare Life Sciences, Piscataway, NJ). Two pairs of oligonucleotides were synthesized for RT-PCR; 5′-TTGGGTTCCAACAAAGAACC-3′ and 5′-CTTTTACAGGGCCTCAGCAG-3′ for *BLID*, and 5′-GGCGACATCAGACAGACAGA-3′ and 5′-AGGACCAGCTGAAAGGAACA-3′ for *LOC399959*. Expression of glyceraldehyde-3-phosphate dehydrogenase (*GAPDH*) was tested for cDNA quantification using 5′-GACAACAGCCTCAAGATCATCA-3′ and 5′-GGTCCACCACTGACACGTTG-3′. PCR reactions were performed under the following condition: initial denaturation at 96°C or 2 minutes, followed by 35 cycles (for *BLID* and *LOC399959*) or 18 cycles (for *GAPDH*) at 96°C for 20 seconds, 60°C for 40 seconds, and polymerization at 72°C for 40 seconds.

## Supporting Information

Table S1 Summary results for the 29 SNPs significant at the *P*<10^−4^ level after population stratification adjustment in the first stage of the genome-wide association analysis.
(0.06 MB XLS)
